# Evaluation of Choroidal Thickness Using Optical Coherent Tomography: A Review

**DOI:** 10.3389/fmed.2021.783519

**Published:** 2021-12-03

**Authors:** Rui Xie, Bingjie Qiu, Jay Chhablani, Xinyuan Zhang

**Affiliations:** ^1^Beijing Institute of Ophthalmology, Beijing Tongren Eye Center, Tongren Hospital, Capital Medical University, Beijing, China; ^2^Beijing Retinal and Choroidal Vascular Diseases Study Group, Beijing, China; ^3^The University of Pittsburgh Medical Center Eye Center, University of Pittsburgh, Pittsburgh, PA, United States

**Keywords:** choroidal thickness, optical coherence tomography, swept-source optical coherence tomography, methodology, morphological investigation

## Abstract

The choroid is the main source of blood and nourishment supply to the eye. The dysfunction of the choroid has been implicated in various retinal and choroidal diseases. The identification and in-depth understanding of pachychoroid spectrum disorders are based on the tremendous progress of optical coherence tomography (OCT) technology in recent years, although visibility of choroid is challenging in the era of the time or spectral domain OCT. The recent rapid revolution of OCTs, such as the enhanced depth imaging OCT and the swept-source OCT, has greatly contributed to the significant improvement in the analysis of the morphology and physiology of the choroid precisely, especially to the choroid–scleral boundary and vasculature. The present review highlights the recently available evidence on the measurement methodology and the clinical significance of choroidal thickness in retinal or choroidal disorders.

## Introduction

The choroid is mainly composed of blood vessels and is the posterior portion of the uveal tract with rich and slow blood flow. As the main source of blood supply to the retinal epithelium, the outer retina, and the optic nerve, the choroid plays a significant role in maintaining the normal metabolism of the retinal pigment epithelium (RPE) and photoreceptors (1). The dysfunction of the choroid has been implicated in various retinal and choroidal diseases. Additionally, choroidal thickness (ChT) is a sensitive biomarker in the prediction, diagnosis, intervention, and follow-up of various acute or chronic retinal and choroidal diseases, including polypoidal choroidal vasculopathy (PCV), central serous chorioretinopathy (CSCR), and idiopathic macular hole (IMH) (2–4).

Optical coherence tomography (OCT) is a non-invasive fundus imaging modality, which plays a vital role in revealing the pathogenesis and development of retinal–choroidal diseases. Compared to other imaging modalities, OCT has greatly improved clinical diagnosis and research since its inception in the 1990s. Furthermore, the wide use of OCT-angiography boosts the OCT field from structural imaging to vascular imaging and provides an opportunity for the quantitative analysis of both ocular structure and vasculature, especially the choroid thickness. Compared to the traditional methods such as ultrasound and indocyanine green angiography (ICGA), the advantage of OCT is non-invasive and repeatable with a higher resolution (5, 6).

Choroidal thickness measuring *in vivo* has been reported in various diseases using different available methods/devices including ultrasound and OCT since 1979 (7, 8). To date, ChT has become a vital predictive imaging biomarker for both retinal and choroidal disorders. In-depth understanding of pathogenesis of these disorders drives us to understand the standard method to measure ChT.

Presently, there is no unified international protocol for ChT measurement by OCT, the most popular methods being manual and automatic segmentation. In this review, we have highlighted the recently available evidence on the measurement methodology of ChT, and the significance of ChT in various retinal or choroidal disorders.

## Anatomical Characteristics of the Choroid

**Figure 1:**
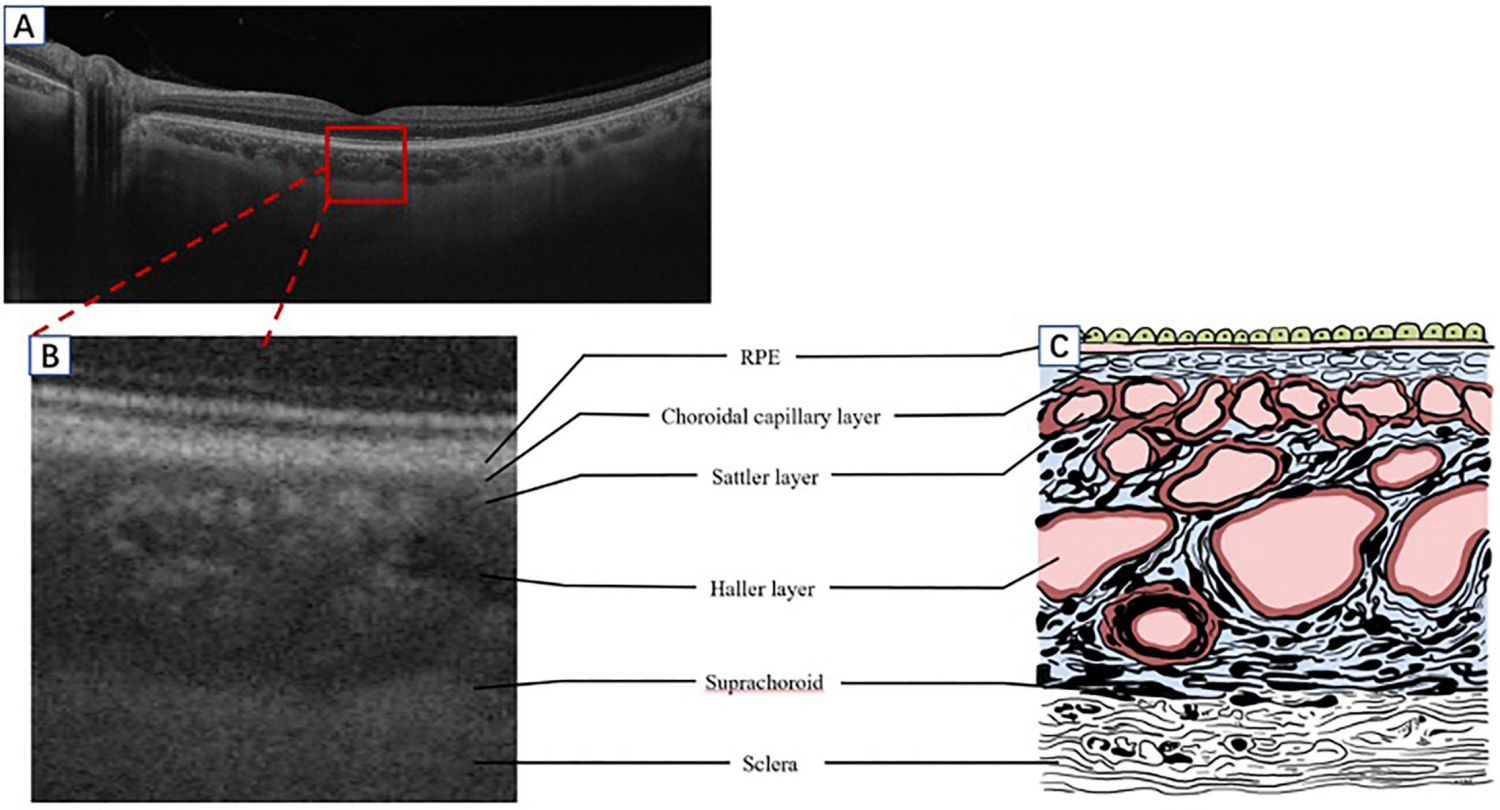
A schematic diagram of the human choroid and the corresponding OCT signal. **(A)** Segmentation of the retina and choroid as shown by SS-OCT B-scan (Plex Elite 9000, Carl Zeiss Meditec, Inc., Oberkochen, Germany). **(B)** A partially enlarged image of the OCT B-scan signal corresponding with a schematic diagram **(C)**. OCT, optical coherence tomography; SS-OCT, swept-source OCT; RPE, retinal pigment epithelium.

The choroid is located between the retina and the sclera. Anatomically, from inside toward outside, it has been described as five layers: Bruch's membrane (BrM), the choroidal capillary (CC), the Sattler, the Haller layers, and the suprachoroid cavity. Approximately 90% of the ocular blood perfusion is supplied by the choroid, 70% of which is from the CC layer. The CC layer has high blood flow, high vascular density, and abundant interstices that play a significant role in the metabolism of photoreceptor cells and the RPE (9). The choroidal blood vasculature is distributed in a leaflet shape and arranged into layers. As the choroidal blood vessel is the only source of nourishment supply for the fovea, the sub-macular choroid is the thickest part. The large vessel calibers of the Haller layer run parallel with its branch in a fan shape, forming a network of lobular capillaries, which communicate with each other and supply blood in different regions. The function of these anastomoses is to shunt blood to balance the circulation pressure of the lobules, and it is the basis for maintaining the function of the retina (9–11). In addition to the blood supply, choroid tissue has various functions, including temperature regulation and the absorption of light to form a dark chamber (12) (Figure 1).

## Principle of Choroid Imaging by OCT

As a non-invasive fundus imaging technology, OCT works by dividing the monochromatic light into two beams through the coupler: the reference arm and the measuring arm, which penetrates the intraocular refractive stroma to the surface of the retina. The two beams are reflected by a mirror, and the fundus tissue could be assessed, respectively. After being combined with the coupler from the interference, the beams were detected by the photodetector based on the heterogeneity and different depths of the tissue. The two or three-dimensional structure images of the biological tissue can be obtained by collecting the varied reflected interference signal (13).

Time domain (TD)-OCT realizes axial scanning (A scan) by reference to the rapid changes in the optical delay lines generated by the mechanical motion of the reference arm, but the scanning depth is limited, and the speed is slow (14, 15). The reference arm of the spectral domain (SD)-OCT is fixed, the interference signal data is recorded through the spectrometer receiver and assessed by Fourier (inverse) transform to obtain the axial depth information, thus greatly improving the speed and depth of the OCT (16). Nevertheless, due to the attenuation and scattering of light by the RPE, TD-OCT, and SD-OCT cannot delineate the choroid details and choroidal scleral interface, rendering it challenging to achieve a high-resolution tomography of the choroid. In addition, Spaide et al. developed an enhanced depth imaging (EDI)-OCT in 2008, which can measure ChT for the first time by moving the zero delay line toward the choroid to present the choroid details of the structure (17). With the development of laser technology, swept-source (SS)-OCT emerged, which improved the imaging speed, depth, software algorithm, and eye-movement tracking technology. This increases the tissue resolution and image signal-to-noise ratio and contributed to an in-depth understanding of choroidal diseases (18–20).

However, compared to ICGA, OCT can only detect the lesion in a static state. Various blood flows are graded to interpret the active blood flow lesions in the dynamic state and quantitate the blood signal, which would be the trend in the future.

### EDI-OCT and the Choroid

**Figure 2:**
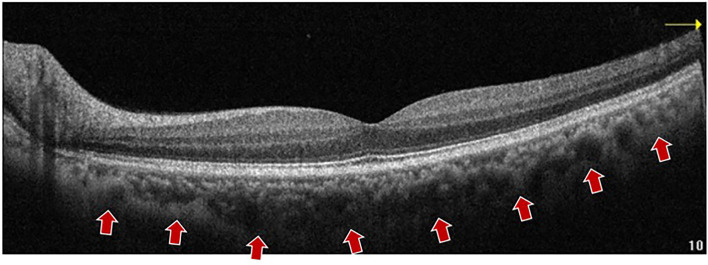
A representative EDI-OCT B-scan imaging of a 57 years old subject. The choroid boundary is detected vaguely. An EDI-OCT (Optovue, Inc., California, United States) B-scan image shows the choroid–sclera boundary (arrows) with a central wavelength of 850 nm, which made the zero-delay line to the choroid by moving the device closer to the eye, thus improving image resolution and facilitating the identification of the choroid–scleral interface. EDI OCT, enhanced depth imaging OCT; SD-OCT, spectral domain OCT.

Conventional SD-OCT uses a high-resolution spectrophotometer to separate the wavelengths due to the zero-delay line at the posterior vitreous boundary level and the light scattering of the RPE layer, making the obtained tomographic images unable to identify the choroid–scleral interface clearly. EDI-OCT proposed by Spaide for the first time in 2008 (17), with a central wavelength of 850 nm, made the zero-delay line to the choroid by moving the device closer to the eye, thus improving image resolution and facilitating the identification of the choroid–scleral interface. In addition, the image averaging technique could increase the signal-to-noise ratio and reduce speckle for enhanced choroid visualization (21, 22) (Figure 2).

### SS-OCT and the Choroid

**Figure 3:**
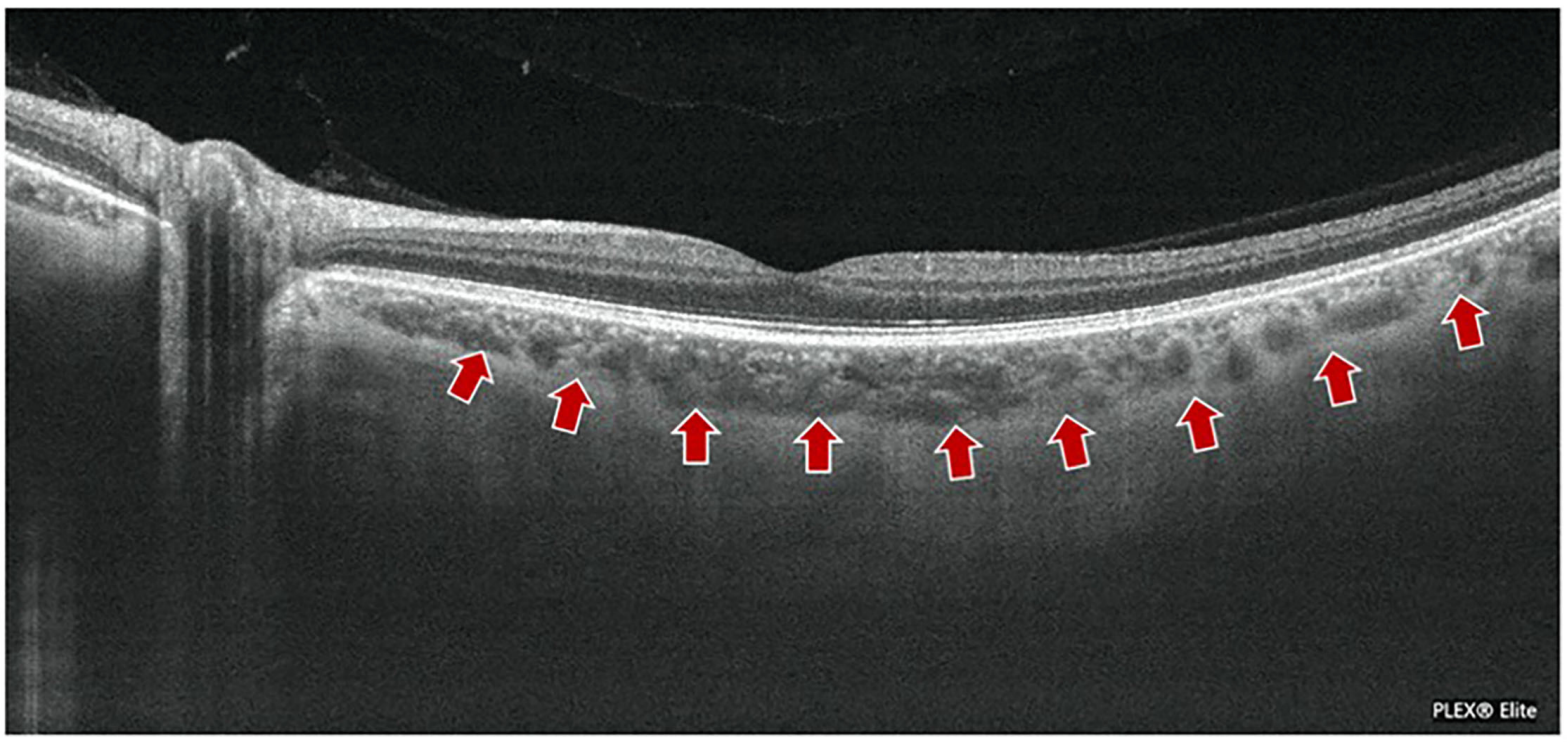
A representative imaging of SS-OCT in normal subject. The boundary of choroid–scleral (arrows) and choroidal vasculature in a 49 year-old man are clearly shown using an SS-OCT (Plex Elite 9000, Carl Zeiss Meditec, Inc., Oberkochen, Germany). SS-OCT could reach up to 6 mm depth, 200,000 A scans per second (Plex Elite 9000) with 200,000 A scans per second, and 6.3 μm of axial resolution.

Compared to SD-OCT, SS-OCT uses a light source with a longer wavelength and a double-balanced light detector. The central wavelength is 1,050 nm/1,060 nm, with strong penetration, and hence is less affected by the light scattering of RPE, lens turbidities, and less signal attenuation, giving a better visualization of the deep layer (23–25). SS-OCT could reach up to 6 mm of depth, 200,000 A scans per second (Plex Elite 9000, Carl Zeiss Meditec, Inc., Oberkochen, Germany), and 6.3 μm of axial resolution (26). After technical innovation of SS-OCT in microelectronics mechanical systems, tunable filter technology, and vertical-current surface-emitting laser, an intensive scanning mode and greater scanning area are realized. Beyond that, the eye movement artifact could be reduced to improve the quality of imaging and make faster imaging inspection with higher efficiency and a wide scanning range (27–29) (Figure 3).

## Methods for Measuring ChT

### Histopathological and Ultrasound

The ChT measured by histopathology is objective but thinner than the true value with respect to the choroid, which is a highly vascularized tissue and ChT varying with its blood perfusion. Moreover, histological fixation can lead to the deformation and shrinkage of the choroid, and the correlation between the measured value and the true value cannot be quantified accurately (30, 31). ICGA is a traditional method for assessing the morphology of choroidal vessels but could not provide anatomical tomography images.

The resolution of ultrasound is lower than that of OCT for choroidal tomography. Ultrasound at 20 MHz achieves posterior imaging, which is mainly used in the case of refractive media opacity and can evaluate the deeper structure (32).

### Optical Coherence Tomography

Presently, there is no international unified reference standard for OCT measurement of ChT. The mainstream methods are mainly divided into manual single-point or multi-point measurement and automatic segmentation methods (Table 1).

**Table 1:** Methods for measuring choroidal thickness.

**Methods for measuring choroidal thickness**		**References**	**Description of measurement, location, and method**
Manual measurement	Subfoveal choroidal thickness (SFCT)	(33)	From the Bruch's membrane to the sclerochoroidal interface at fovea.
		(34)	At the fovea with enhanced depth imaging OCT, 9-mm horizontal and vertical scans through the foveal center.
	Three-point method	(6)	From the outer edge of the hyper-reflective RPE to the sclerochoroidal interface at the fovea, 750 μm temporal to the fovea, and 750 μm nasal to the fovea.
		(35)	From the outer edge of the hyper-reflective RPE to the sclerochoroidal interface at the fovea and at 1,000 μm nasal and temporal to the fovea.
		(36)	
	Five-point method	(37)	The vertical distance from the Bruch's membrane to the sclerochoroidal interface at fovea and 1,500 μm nasally, 1,500 μm superiorly, 1,500 μm temporally, and 1,500 μm inferiorly apart from the foveal center.
		(38)	At the fovea and 3,000 μm nasal, temporal, superior, and inferior to the fovea in the horizontal and vertical sections.
	Seven-point method	(39)	At the fovea and 500 μm, 1,000 μm and 1,500 μm nasal and temporal to the fovea.
	Nine-point method	(40)	The vertical distance from the Bruch membrane to the sclerochoroidal interface at fovea, and nasal respective temporal at 500 μm, 1,000 μm, 1,500 μm and 2,000 μm distance from the fovea.
		(41)	At the fovea and 1,000 μm and 3,000 μm to the fovea superiorly, inferiorly, temporally, and nasally.
		(42)	At the fovea and 1,500 μm and 3,000 μm from the center of the fovea in areas of superior, temporal, inferior, and nasal quadrants.
	Macular choroidal thickness	(43)	The fovea and 1,000 μm intervals from the fovea to a distance of 3,000 μm in the nasal, temporal, superior, and inferior directions. The average of 14 choroidal thickness readings was recorded as the macular choroidal thickness.
Automatic measurement		(3) (44) (45) (46)	The choroidal thickness was automatically measured with choroidal thickness map using the Early Treatment Diabetic Retinopathy Study grid (EDTRS). It's divided into 9 sectors in the grid. The diameters for central foveal circle, parafoveal circle, and perifoveal circle were 1, 3, and 6 mm, respectively.

#### Manual Method

**Figure 4:**
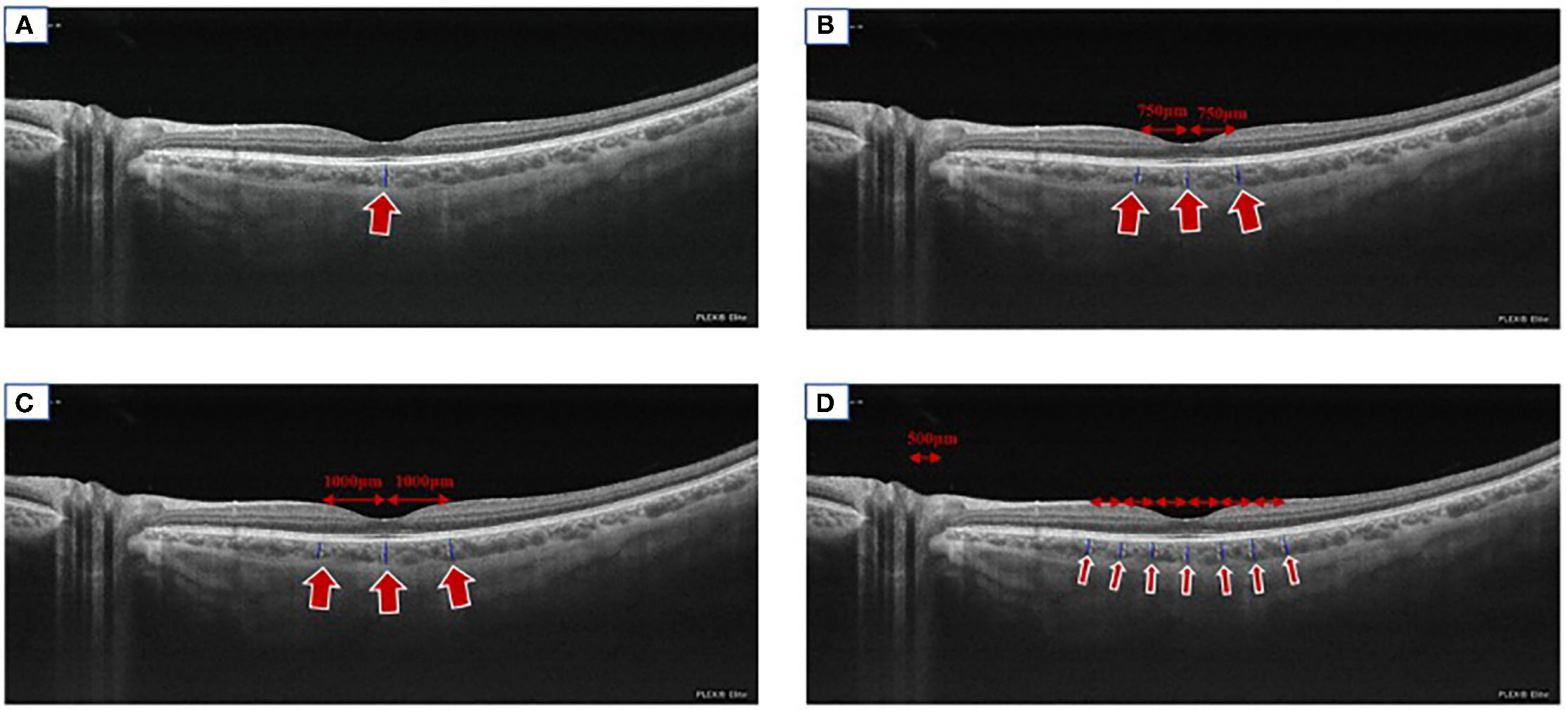
Manual measuring methods. A representation of imaging showing the routinely used manual measuring methods. **(A)** We took SS-OCT (Plex Elite 9000, Carl Zeiss Meditec, Inc., Oberkochen, Germany) as an example to display the single-point method (arrow). **(B)** The three-point method (the fovea, at 750 nasally to the fovea and temporal to the fovea, arrows). **(C)** The three-point method (the fovea, 1,000 μm nasally to the fovea and temporally to the fovea, arrows). **(D)** The seven-point method (the fovea, 500, 1,000, and 1,500 μm nasally and temporally to the fovea respectively, arrows).

Manual methods have been described as single-point and multi-point methods. Theoretically, subfoveal choroidal thickness (SFCT) is the thickest part of the choroid, but single-point measurement cannot reflect the overall information of the choroid (47). The multi-point methods were further categorized into horizontal and vertical techniques. The horizontal multi-point method was utilized to measure the SFCT, the nasal and temporal sides of the fovea. The multipoint can be three points, seven points, and nine points. The three points are located at 750 μm and 1,000 μm to the fovea temporally and nasally, the seven points and nine points are located at 500 μm intervals in the horizontal section (6, 35, 36, 39, 40). Some other multi-point methods simultaneously measure the ChT horizontally and vertically. Five or nine points are placed at the SFCT and its superior, inferior, nasal, and temporal sides. The five points are located at the 1,500 μm or 3,000 μm to the fovea superiorly, inferiorly, temporally and nasally. The nine-points are located at the 1,000 μm, 3,000 μm or with the 1,500 μm interval to the fovea superiorly, inferiorly, temporally and nasally (37, 38, 41, 42). Although manual measurement has good repeatability (48), it is difficult to avoid errors. The multipoint and multi-quadrant measurement reflects the average ChT, the distribution, and trend of ChT in cohort population and reduces the single-point error (Figure 4).

#### Automatic Segmentation Method

**Figure 5:**
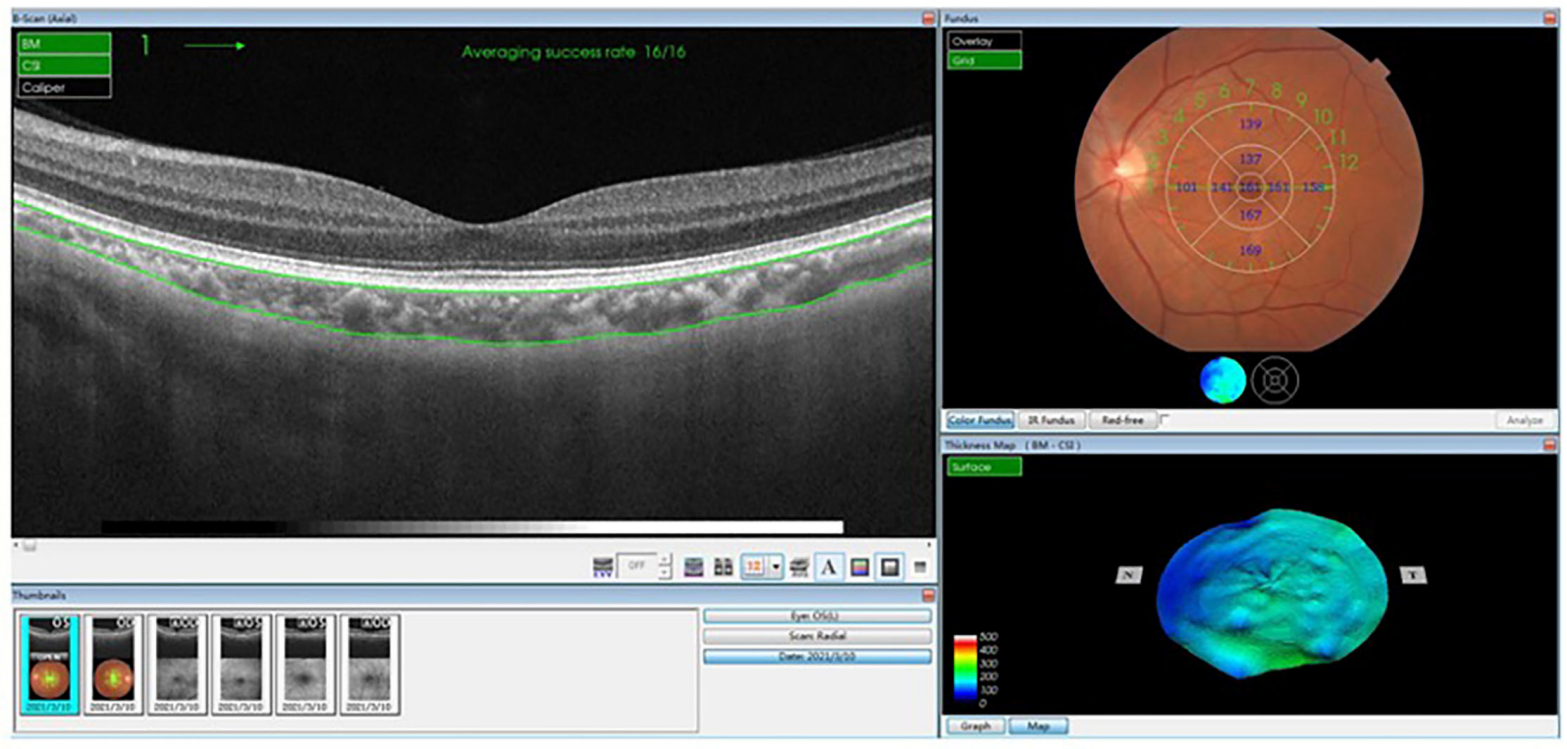
The ETDRS automatic segmentation method using TOPCON advanced boundary segmentation-TABS software. An ETDRS map is generated automatically, which can be corrected manually. The ETDRS grid is composed of three concentric circles: the diameter of the fovea, parafovea, and perifovea are 1, 3, and 6 mm, respectively. The average choroidal thickness in the central circular and the eight sectors of superior, inferior, nasal, and temporal areas can be calculated, respectively. ETDRS, Early Treatment Diabetic Retinopathy Study.

The choroid is segmented scanned by EDI-OCT (Heidelberg Spectralis, Heidelberg Engineering, Heidelberg, Germany) and SS-OCT (Triton DRI OCT, Topcon, Tokyo, Japan) through the Early Treatment Diabetic Retinopathy Study (ETDRS) grid using the Heidelberg Engineering software and the TOPCON Advanced Boundary Segmentation-TABS software (49, 50). Each image is constituted by the average of 32 overlapping continuous scans, covering an area of 12 mm × 9 mm with 12 radial scans, providing a three-dimensional ChT mapping by measuring ChT at any point in the macula (51). The reference line is adjusted from the retinal boundary (the internal limiting membrane—RPE) to the choroid boundary (the RPE–choroid–scleral junction), and then an ETDRS map is generated automatically, which can be corrected manually. The ETDRS grid is composed of three concentric circles: the diameter of the fovea, parafovea, and perifovea are 1, 3, and 6 mm, respectively. The average ChT in the central circular and the eight sectors of the nasal inner macula, superior inner macula, temporal inner macula, inferior inner macula, nasal outer macula, superior outer macula, temporal outer macula, and inferior outer macula areas can be calculated, respectively (44–46, 52, 53) (Figure 5).

## Choroidal Thickness and its Influencing Factors

The majority of the studies measured the ChT as the height from the BrM to the choroid–scleral interface (33, 37, 40, 54, 55); however, some studies have defined it as the height from the lower boundary of the hyperreflective RPE to the choroid–scleral interface (34, 38, 42, 43, 56). Margolis and Spaide (57) pointed out that subfoveal CT (SFCT) is the thickest part in the average age of 50.4-years among the 54 normal eyes in a retrospective study, and the average SFCT was 287 ± 76 microns by using Spectralis OCT (Spectralis, Heidelberg Engineering Co, Germany). Subsequently, ChT is rather thin from subfoveal to peripheral retina, temporal thicker than nasal, upward than downward, and peri-optic papilla is the thinnest, which is associated with choroidal vein distribution (58). Interestingly, the results of the study by Ikuno and Ruiz-Moreno are similar (59, 60).

As one of the objective biomarkers for the evaluation of choroid, ChT is dependent on the physiological and pathological factors of the body, and varies with age, refractive, axial length, or diurnal variation (17, 61, 62). A large number of studies have confirmed that age and axial length are the primary factors influencing ChT, and both are negatively correlated with ChT (63–65). Spaide et al. pointed out that ChT decreases 15 μm per 10 years (17). In a longitudinal study based on 3,233 Chinese individuals, SFCT was reported to be 254 ± 107 μm (Spectralis, Heidelberg Engineering Co) with an average age of 65 years (55). Lee et al. reported that SFCT was [median (IQR): 370] 312–406 μm (Spectralis, Heidelberg Engineering Co.) in 741 young adults aged 19–30 years (66).

In addition, the normal ChT differs among different measuring instruments. Bhayana (63) reported that the ChT measured by SS-OCT (DRI-OCT Triton Plus, Topcon, Tokyo, Japan) was slightly higher than that of SD-OCT (Spectralis, Heidelberg Engineering Co.), which was consistent with the results of Matsuo and Copete. This could be attributed to the ability of SS-OCT to identify the choroid–scleral interface precisely (67–69).

Moreover, it is yet to be elucidated whether cardiovascular risk factors have an impact on the choroid as it is a vascular tissue. In a case–control study, Schuster et al. (40) demonstrated that SFCT was associated with the left ventricular end-diastolic blood pressure, systolic blood pressure, epidermal growth factor receptor, and dyslipidemia. By logistic analysis, it was suggested that this correlation is associated with age. Thus, additional qualitative and quantitative studies are warranted to provide more clinical evidence of ChT.

## Clinical Significance of ChT

The choroid provides oxygen and nutrition to the outer five layers of the retina and plays a vital role in the metabolism of RPE and photoreceptors. Therefore, its pathological state is closely related to several diseases, especially pachychoroid spectrum diseases (PCDs), age-related macular degeneration (AMD), and choroidal atrophy associated with pathological myopia. Choroidal vasodilation and/or hyper-perfusion and elevated hydrostatic pressure lead to increased choroidal permeability and focal choroidal exudation with ChT thickening (70). While hypoperfusion and ChT thinning lead to metabolic disorders of RPE and photoreceptor, the growth factors secreted by RPE are insufficient to maintain the physiological needs of the choroidal vessels in reverse, which form a vicious circle and accelerate the progress of the disease (71). Therefore, ChT can be used as a quantitative index to evaluate and reveal the pathophysiology of choroidal diseases.

### Diseases With Increased ChT

#### Pachychoroid Spectrum Diseases

Pachychoroid spectrum disease is a new entity in recent years, which is characterized by the thickening of choroidal layers and dysfunction of choroidal vasculature. PCD usually exhibits chronic continuous thickening and abnormal expansion of the vasculature in the Haller layer and compression of the Sattler and CC layers. The dysfunction of the RPE–BrM–CC complex, intralobular congestion, and stasis may produce an ischemic microenvironment, promote vascular endothelial growth factor (VEGF) expression, leading to secondary choroidal neovascularization (72). Presently, PCD comprises seven major diseases: CSCR, pachychoroid pigment epitheliopathy, pachychoroid neovasculopathy, PCV/aneurysmal type 1 neovascularization, peripapillary pachychoroid disease, and focal choroidal excavation (73).

Polypoidal choroidal vasculopathy is a choroidal vascular disease characterized by polypoidal lesions with diffuse or local dilation of choroidal vascular endings and an abnormal branch vascular network. In a cross-sectional study, Jordan-Yu et al. investigated 100 treatment-naïve PCV eyes, and they found that total lesion areas and polypoidal lesion areas tend to be larger in eyes with increasing SFCT (74), indicating that the pathological features of choroidal background might be the predictive factors influencing the phenotype or progression of PCV. Furthermore, choroidal thickening is a major risk factor in the pathogenesis of PCV, which is a strong evidence differentiated from wet AMD.

Central serous chorioretinopathy is characterized by serous detachment of the neurosensory retina secondary to one or more focal lesions of the RPE. In a prospective study, 34 patients with CSCR in 44 eyes were assessed by SS-OCT. The average ChT in the choroidal leakage area in fluorescein fundus angiography was significantly greater than that in the non-leakage area, and the average ChT in the high permeability region in ICGA was significantly larger than that in the non-pathological area, indicating that increase of hydrostatic pressure in the local choroidal region contributes to CSCR (3). Furthermore, ChT plays a major role in the follow-up of CSCR, which predicts the recurrence or progression of CSCR (75, 76). Half-dose or half-fluence photodynamic therapy reduces SFCT, but patients with a thicker choroid are more likely to have recurrence CSCR (77, 78).

#### Peripheral Exudative Hemorrhagic Chorioretinopathy

Peripheral exudative hemorrhagic chorioretinopathy (PEHCR), also known as eccentric degeneration, extramacular disciform degeneration, and peripheral AMD, is characterized by peripheral subretinal or sub-RPE hemorrhages and/or exudation or peripheral neovascularization in elderly patients. PEHCR primarily affects the neurosensory retina or RPE and the lesions are mostly located at the temporal side of the retina (79). PEHCR and PCV share some mutual clinical and pathophysiological features such as serous or hemorrhagic pigment epithelium detachment, lipid exudation, and abnormal choroid branching vascular network visible on ICGA or OCTA, suggesting that PEHCR may be a peripheral subtype of PCV (80). Yorihisa et al. (81) documented that PEHCR has choroidal vascular alterations resembling PCV in the affected area on ICGA in a 74-year-old patient. In a retrospective, observational, comparative case series, the choroid was significantly thicker in temporal periphery in PEHCR eyes than that in control eyes (272.70 ± 80.20 μm vs. 166.60 ± 40.10 μm, *p* = 0.0002), the mean large choroidal vessel thickness is significantly thicker in PEHCR eyes in comparison with the control eyes (202.40 ± 50.80 μm vs.160.60 ± 40.50 μm, *p* = 0.0235), suggesting the favor inclusion of PEHCR in the pachychoriod disease spectrum (82). Larger cohort studies are warranted to further understand the pathophysiology of PEHCR and to investigate whether PEHCR is a new entity of PCD.

#### Vogt–Koyanagi–Harada Disease

Vogt—Koyanagi–Harada (VKH) is autoimmune granulomatous inflammatory disorder, involving bilateral eyes. Studies have shown that the ChT increased in the acute stage of VKH while it decreased in the recovery period (83, 84). Nakayama (85) demonstrated that the subclinical manifestations were >100 μm ChT with recurrent VKH before ocular inflammation; therefore, ChT could be used as an indicator for the acute deterioration of inflammation. In patients with chronic VKH (average course of disease 106.3 months), Jap et al. (86) found that the mean SFCT (272.38 ± 118.72 μm) in the active period verified by ICGA was significantly higher than that in the quiet period (187.31 ± 109.90 μm) (*P* = 0.002), SFCT could be used to monitor the activity of chronic VKH. However, continuously thinning ChT was observed in patients with long-term chronic VKH (36).

### Diseases With Decreased ChT

#### Age-Related Macular Degeneration

Wet AMD is characterized by the formation of the macular neovascularization (MNV), dry form is characterized by atrophy of RPE and photoreceptors. Moderate-sized drusen (>63 μm in diameter) and RPE changes were the early stage of pathologic changes, proceeding with geographic atrophy and MNV in the late stage. Clinical studies have shown that ChT in patients with advanced AMD was thinner than that of normal controls, while it did not differ from that of normal individuals with early AMD (87–89). Decreased ChT reflects the degree of choroidal atrophy, suggesting that the development of AMD is related to the involvement and loss of microvessels, which is an indicator in the differential of PCV (37).

The occurrence and development of MNV are accompanied by choroidal thickening in the corresponding local regions, and thus, regular measurement of ChT is valuable in predicting MNV formation (90). Furthermore, SFCT could be used as an independent predictor of prognosis of wet AMD, the thicker the choroid is, the more anti-VEGF intervention is needed (91).

#### Pathological Myopia

Several studies have confirmed that the decrease in ChT is related to the degree of refraction. SFCT becomes thinner with the increase in diopter and the lengthening of the ocular axis, accompanied by the thinning of the sclera (92–94). It has been reported that SFCT decreased by 15 μm for every 1 D increase in the myopia diopter, and 32 μm for every 1 mm increase in the axial length in the range of myopia diopter above −1.00 D (55). Ho et al. (56) reported that the average SFCT (118 ± 68 μm) was negatively correlated with diopter in a cross-sectional study of 56 myopic patients (average diopter −8.7 D); for every 1 D increase, SFCT decreased by 6.205 μm; and for every 10 μm increase in SFCT, visual acuity improved by 0.02 LogMAR visual acuity. Choroidal thinning is correlated with the pathogenesis of myopic fundus lesions. A 2-year prospective study revealed that SFCT is an independent predictor of the progression of myopic macular disease (95). With the increase in diopter and axial length, ChT becomes thinner, proceeded with decreased retinal blood supply leading to secondary pathological changes such as lacquer cracks and MNV-related maculopathy.

#### Idiopathic Macular Hole

Idiopathic macular hole is characterized by retinal neuroepithelial tissue defect in the fovea, which is the most common type of macular hole. IMHs are more common in females than in males and usually manifest in 55 years or older adults. In a cross-sectional study of 50 patients with IMH, Zeng et al. (41) revealed that the SFCT of IMH (206.82 ± 67.09 μm) was significantly thinner than that of the controls (248.88 ± 63.10 μm) (*P* = 0.002), and the SFCT of contralateral eyes (228.34 ± 80.71 μm) was thinner than that of controls, albeit not significantly. The results of the studies by Zhang and Reibaldi were consistent, suggesting that choroidal perfusion may play a vital role in the pathogenesis of IMH. The contralateral eye with thinner ChT was also prone to IMH, such that routine follow-up is necessary (4, 96). However, Sogawa pointed out that SFCT did not have a significant correlation with total choroidal blood flow and subfoveal choroidal blood flow (97). In conclusion, except for the traction mechanism proposed by Gass (98), the significant thinning of ChT may be related to the ischemic degeneration of the outer retina, which might be crucial for the occurrence and development of IMH.

#### Other Systemic Diseases

Craig et al. found that decreased ChT in chronic kidney disease correlated with lower estimated glomerular filtration rate and severe proteinuria, concomitated with the increased expression level of IL-6, C-reactive protein, and endothelin-1 (99). It was also found that endothelial dysfunction markers (endothelin-1, von Willebrand factor) were negatively correlated with ChT in cirrhosis patients (100), indicating that the morphological changes of the choroid are mediated by inflammatory diseases. Further well-designed randomized clinical trials are warranted to investigate whether ChT can be used as an auxiliary biomarker for systemic disorders. In addition, the thinner choroid in patients with Alzheimer's disease was found, which may be associated with vascular wall deposition of amyloid beta-protein (101). Similarly, whether ChT could be used as an early biomarker of Alzheimer's disease to predict disease progression is yet to be confirmed.

## Conclusion

Currently, EDI SD-OCT and SS-OCT could be used clinically due to its non-invasive, high-resolution, cross-sectional imaging characteristics of the choroid and measuring the ChT with good repeatability (6, 102). To date, there is no unified standard for ChT measurement that is carried out manually or via automatic segmentation method according to the ETDRS grid. Thus, methods for ChT measurement need to be standardized to objectively reflect the choroidal structure and reveal its clinical significance in the occurrence and development of the diseases. The establishment of ChT normal value needs large-scale, multicenter research to explore the pathogenesis of the fundus diseases and provide a new horizon on the management. In addition, whether ChT could quantitatively reflect the choroidal blood flow needs further study. The eye could be regarded as a monitoring window of systemic diseases and an auxiliary biomarker of the eye or systemic diseases, which plays a vital role in the diagnosis, management, and follow-up of diseases.

## Author Contributions

XZ contributed to the conception and revised the manuscript. RX drafted the manuscript. BQ helped to draft the manuscript. JC provided comments and revised the manuscript. All authors contributed to the manuscript revision, read, and approved the submitted version.

## Funding

This work was supported by the National Natural Science Foundation of China [Grant Nos: 81570850, 81170859, and 82070988] and the Ministry of Science and Technology Foundation of China [Grant No: 2016YFC1305604].

## Conflict of Interest

The authors declare that the research was conducted in the absence of any commercial or financial relationships that could be construed as a potential conflict of interest.

## Publisher's Note

All claims expressed in this article are solely those of the authors and do not necessarily represent those of their affiliated organizations, or those of the publisher, the editors and the reviewers. Any product that may be evaluated in this article, or claim that may be made by its manufacturer, is not guaranteed or endorsed by the publisher.

## References

[1] Linsenmeier R Padnick-Silver L. Metabolic dependence of photoreceptors on the choroid in the normal and detached retina. Invest. Ophthalmol. Visual Sci. (2000) 4110:3117–23. 10967072

[2] Lee K Park J Park Y Park Y. Analysis of choroidal thickness and vascularity in patients with unilateral polypoidal choroidal vasculopathy. Graefes Arch Clin Exp Ophthalmol. (2020) 2586:1157–64. 10.1007/s00417-020-04620-z 32037487

[3] Jirarattanasopa P Ooto S Tsujikawa A Yamashiro K Hangai M Hirata M. Assessment of macular choroidal thickness by optical coherence tomography and angiographic changes in central serous chorioretinopathy. Ophthalmology. (2012) 1198:1666–78. 10.1016/j.ophtha.2012.02.021 22521082

[4] Zhang P Zhou M Wu Y Lu B Li T Zhao J. Choroidal thickness in unilateral idiopathic macular hole: a cross-sectional study and meta-analysis. Retina. (2017) 371:60–9. 10.1097/IAE.0000000000001118 27322947

[5] Borrelli E Sarraf D Freund K Sadda S. OCT angiography and evaluation of the choroid and choroidal vascular disorders. Progr Retinal Eye Res. (2018) 67:30–55. 10.1016/j.preteyeres.2018.07.002 30059755

[6] Branchini L Regatieri C Flores-Moreno I Baumann B Fujimoto J Duker J. Reproducibility of choroidal thickness measurements across three spectral domain optical coherence tomography systems. Ophthalmology. (2012) 1191:119–23. 10.1016/j.ophtha.2011.07.002 21943786 PMC3251715

[7] Coleman D Lizzi F. *In vivo* choroidal thickness measurement. Am J Ophthalmol. (1979) 88:369–75. 10.1016/0002-9394(79)90635-4 484666

[8] Hillenkamp J Hussain A Jackson T Cunningham J Marshall J. The influence of path length and matrix components on ageing characteristics of transport between the choroid and the outer retina. Invest Ophthalmol Visual Sci. (2004) 455:1493–8. 10.1167/iovs.03-0765 15111607

[9] Bill A Sperber G Ujiie K. Physiology of the choroidal vascular bed. Int Ophthalmol. (1983) 62:101–7. 10.1007/BF00127638 6403480

[10] Yoneya S Tso M. Angioarchitecture of the human choroid. Arch Ophthalmol. (1987) 1055:681–7. 10.1001/archopht.1987.01060050099046 3619746

[11] Olver J. Functional anatomy of the choroidal circulation: methyl methacrylate casting of human choroid. Eye. (1990) : 262–72. 10.1038/eye.1990.38 2379644

[12] Nickla D Wallman J. The multifunctional choroid. Progr Retinal Eye Res. (2010) 292:144–68. 10.1016/j.preteyeres.2009.12.002 20044062 PMC2913695

[13] Aumann S Donner S Fischer J Müller F. Optical Coherence Tomography (OCT): principle and technical realization. In: Bille JF, editor. High Resolution Imaging in Microscopy and Ophthalmology: New Frontiers in Biomedical Optics. Cham: Springer Copyright 2019 (2019). p. 59–85. 10.1007/978-3-030-16638-0_3 32091846

[14] Fercher A Mengedoht K Werner W. Eye-length measurement by interferometry with partially coherent light. Opt Lett. (1988) 133:186–8. 10.1364/OL.13.000186 19742022

[15] Forte R Cennamo G Finelli M de Crecchio G. Comparison of time domain Stratus OCT and spectral domain SLO/OCT for assessment of macular thickness and volume. Eye. (2009) 2311:2071–8. 10.1038/eye.2008.363 19079147

[16] Schmitt JM. Optical coherence tomography (OCT): a review. IEEE J Selected Top Quant Electron. (2002) 54:1205–15 10.1109/2944.79634831957060

[17] Spaide R Koizumi H Pozzoni M Pozonni M. Enhanced depth imaging spectral-domain optical coherence tomography. Am J Ophthalmol. (2008) 1464:496–500. 10.1016/j.ajo.2008.05.032 18639219

[18] Sainter AW King TA Dickinson MR. Effect of target biological tissue and choice of light source on penetration depth and resolution in optical coherence tomography. J Biomed Opt. (2004) 91:193–9. 10.1117/1.1628243 14715073

[19] Marschall S Pedersen C Andersen PE. Investigation of the impact of water absorption on retinal OCT imaging in the 1060 nm range. Biomed Opt Express. (2012) 37:1620. 10.1364/BOE.3.001620 22808433 PMC3395486

[20] Itakura H Kishi S Li D Akiyama H. En face imaging of posterior precortical vitreous pockets using swept-source optical coherence tomography. Invest Ophthalmol Vis. (2015) 565:2898–2900. 10.1167/iovs.15-16451 26029885

[21] Spaide R Ledesma-Gil G. Novel method for image averaging of optical coherence tomography angiography images. Retina. (2020) 4011:2099–105. 10.1097/IAE.0000000000002877 32604340

[22] Mrejen S Spaide R. Optical coherence tomography: imaging of the choroid and beyond. Survey Ophthalmol. (2013) 585:387–429. 10.1016/j.survophthal.2012.12.001 23916620

[23] Mansouri K Medeiros F Marchase N Tatham A Auerbach D Weinreb R. Assessment of choroidal thickness and volume during the water drinking test by swept-source optical coherence tomography. Ophthalmology. (2013) 12012:2508–16. 10.1016/j.ophtha.2013.07.040 24021895 PMC3833954

[24] Ferrara D Mohler KJ Waheed N Adhi M Liu JJ Grulkowski I. En Face enhanced-depth swept-source optical coherence tomography features of chronic central serous chorioretinopathy. Ophthalmology. (2014) 1213:719–26. 10.1016/j.ophtha.2013.10.014 24289918 PMC3943670

[25] Motaghiannezam R Schwartz D Fraser S. *In vivo* human choroidal vascular pattern visualization using high-speed swept-source optical coherence tomography at 1060 nm. Invest Ophthalmol Vis Sci. (2012) 534:2337–48. 10.1167/iovs.11-7823 22410568

[26] Blatter C Klein T Grajciar B Schmoll T Wieser W Andre R. Ultrahigh-speed non-invasive widefield angiography. J Biomed Opt. (2012) 17:070505. 10.1117/1.JBO.17.7.070505 22894461

[27] Choi WJ Waheed NK Moult EM Adhi M Fujimoto JG. Ultrahigh speed swept source optical coherence tomography angiography of retinal and choriocapillaris alterations in diabetic patients with and without retinopathy. Retina. (2016) 371:11. 10.1097/IAE.0000000000001250 27557084 PMC5177496

[28] Moult EM Waheed NK Novais EA Choi WJ Lee BK Ploner SB. Swept-source optical coherence tomography angiography reveals choriocapillaris alterations in eyes with nascent geographic atrophy and drusen-associated geographic atrophy. Retina. (2016) 36:S2–11. 10.1097/IAE.0000000000001287 28005659 PMC5193240

[29] Yokoi T Toriyama N Yamane T Nakayama Y Nishina S Azuma N. Development of a premacular vitreous pocket. JAMA Ophthalmol. (2013) 1318:1095–6. 10.1001/jamaophthalmol.2013.240 23765255

[30] Anger E Unterhuber A Hermann B Sattmann H Schubert C Morgan J. Ultrahigh resolution optical coherence tomography of the monkey fovea. Identification of retinal sublayers by correlation with semithin histology sections. Exp Eye Res. (2004) 786:1117–25. 10.1016/j.exer.2004.01.011 15109918

[31] Manjunath V Taha M Fujimoto J Duker J. Choroidal thickness in normal eyes measured using Cirrus HD optical coherence tomography. Am J Ophthalmol. (2010) 1503:325–9.e1. 10.1016/j.ajo.2010.04.018 20591395 PMC2926223

[32] Coleman D Silverman R Chabi A Rondeau M Shung K Cannata J. High-resolution ultrasonic imaging of the posterior segment. Ophthalmology. (2004) 1117:1344–51. 10.1016/j.ophtha.2003.10.029 15234135

[33] Bo Q Yan Q Shen M Song M Sun M Yu Y. appearance of polypoidal lesions in patients with polypoidal choroidal vasculopathy using swept-source optical coherence tomographic angiography. JAMA Ophthalmol. (2019) 1376:642–50. 10.1001/jamaophthalmol.2019.0449 30998817 PMC6567849

[34] Koizumi H Kano M Yamamoto A Saito M Maruko I Sekiryu T. subfoveal choroidal thickness during aflibercept therapy for neovascular age-related macular degeneration: twelve-month results. Ophthalmology. (2016) 1233:617–24. 10.1016/j.ophtha.2015.10.039 26686967

[35] Eymard P Gerardy M Bouys L Mehanna C Bertherat J Behar-Cohen F. Choroidal imaging in patients with Cushing syndrome. Acta Ophthalmol. (2020) 995:533–7. 10.1111/aos.14664 33196148

[36] da Silva F Sakata V Nakashima A Hirata C Olivalves E Takahashi W. Enhanced depth imaging optical coherence tomography in long-standing Vogt-Koyanagi-Harada disease. Br J Ophthalmol. (2013) 971:70–4. 10.1136/bjophthalmol-2012-302089 23099292

[37] Chung S Kang S Lee J Kim Y. Choroidal thickness in polypoidal choroidal vasculopathy and exudative age-related macular degeneration. Ophthalmology. (2011) 1185:840–5. 10.1016/j.ophtha.2010.09.012 21211846

[38] Fang Y Du R Nagaoka N Yokoi T Shinohara K Xu X. OCT-based diagnostic criteria for different stages of myopic maculopathy. Ophthalmology. (2019) 1267:1018–32. 10.1016/j.ophtha.2019.01.012 30703442

[39] Govetto A Sarraf D Figueroa M Pierro L Ippolito M Risser G. Choroidal thickness in non-neovascular versus neovascular age-related macular degeneration: a fellow eye comparative study. Br J Ophthalmol. (2017) 1016:764–9. 10.1136/bjophthalmol-2016-309281 27587716

[40] Schuster A Leuschner A Feretos C Blumenstein P Troebs S Schwuchow S. Choroidal thickness is associated with cardiovascular risk factors and cardiac health: the Gutenberg Health Study. Clin Res Cardiol. (2020) 1092:172–82. 10.1007/s00392-019-01498-8 31168641

[41] Zeng J Li J Liu R Chen X Pan J Tang S. Choroidal thickness in both eyes of patients with unilateral idiopathic macular hole. Ophthalmology. (2012) 11911:2328–33. 10.1016/j.ophtha.2012.06.008 22892154

[42] Moschos M Nitoda E Laios K Ladas D Chatziralli I. The impact of chronic tobacco smoking on retinal and choroidal thickness in greek population. Oxidat Med Cell Longevity. (2016) 2016:2905789. 10.1155/2016/2905789 26885247 PMC4738968

[43] Wu W Shih C Wang N Lien R Chen Y Chao A. Choroidal thickness in patients with a history of retinopathy of prematurity. JAMA Ophthalmol. (2013) 13111:1451–8. 10.1001/jamaophthalmol.2013.5052 24077425

[44] Yuan N Li J Tang S Li F Lee C Ng M. Association of secondhand smoking exposure with choroidal thinning in children aged 6 to 8 years: the Hong Kong children eye study. JAMA Ophthalmol. (2019) 13712:1406–14. 10.1001/jamaophthalmol.2019.4178 31621803 PMC6802252

[45] Suh M Zangwill L Manalastas P Belghith A Yarmohammadi A Medeiros F. Deep retinal layer microvasculature dropout detected by the optical coherence tomography angiography in glaucoma. Ophthalmology. (2016) 12312:2509–18. 10.1016/j.ophtha.2016.09.002 27769587 PMC5360450

[46] Shin J Shin Y Lee B. Choroidal thickness and volume mapping by a six radial scan protocol on spectral-domain optical coherence tomography. Ophthalmology. (2012) 1195:1017–23. 10.1016/j.ophtha.2011.10.029 22281089

[47] Lim H Kim K Won Y Lee W Lee M Kim J. A comparison of choroidal thicknesses between pachychoroid and normochoroid eyes acquired from wide-field swept-source OCT. Acta Ophthalmol. (2020) 99:e117–23. 10.1111/aos.14522 32573109

[48] Rahman W Chen F Yeoh J Patel P Tufail A Da Cruz L. Repeatability of manual subfoveal choroidal thickness measurements in healthy subjects using the technique of enhanced depth imaging optical coherence tomography. Invest Ophthalmol Vis Sci. (2011) 525:2267–71. 10.1167/iovs.10-6024 21087970

[49] Wiacek M Machalińska A. Evaluation of choroidal parameters in eyes at the first onset of acute anterior uveitis. BMC Ophthalmol. (2019) 191:63. 10.1186/s12886-019-1072-7 30819128 PMC6396533

[50] Nagasawa T Mitamura Y Katome T Shinomiya K Naito T Nagasato D. Macular choroidal thickness and volume in healthy pediatric individuals measured by swept-source optical coherence tomography. Invest Ophthalmol Vis Sci. (2013) 5410:7068–74. 10.1167/iovs.13-12350 24106114

[51] Esmaeelpour M Povazay B Hermann B Hofer B Kajic V Kapoor K. Three-dimensional 1060-nm OCT: choroidal thickness maps in normal subjects and improved posterior segment visualization in cataract patients. Invest Ophthalmol Vis Sci. (2010) 5110:5260–6. 10.1167/iovs.10-5196 20445110

[52] Fang D Li Q Yan K Xu S Jiang J Che X. Retinal and choroidal thickness in relation to c-reactive protein on swept-source optical coherence tomography. J Immunol Res. (2021) 2021:6628224. 10.1155/2021/6628224 33564690 PMC7850851

[53] Agawa T Miura M Ikuno Y Makita S Fabritius T Iwasaki T. Choroidal thickness measurement in healthy Japanese subjects by three-dimensional high-penetration optical coherence tomography. Graefes Arch Clin Exp Ophthalmol. (2011) 24910:1485–92. 10.1007/s00417-011-1708-7 21556938

[54] Xu J Xu L Du K Shao L Chen C Zhou J. Subfoveal choroidal thickness in diabetes and diabetic retinopathy. Ophthalmology. (2013) 12010:2023–8. 10.1016/j.ophtha.2013.03.009 23697958

[55] Wei W Xu L Jonas J Shao L Du K Wang S. Subfoveal choroidal thickness: the Beijing Eye Study. Ophthalmology. (2013) 1201:175–80. 10.1016/j.ophtha.2012.07.048 23009895

[56] Ho M Liu D Chan V Lam D. Choroidal thickness measurement in myopic eyes by enhanced depth optical coherence tomography. Ophthalmology. (2013) 1209:1909–14. 10.1016/j.ophtha.2013.02.005 23683921

[57] Margolis R Spaide R. A pilot study of enhanced depth imaging optical coherence tomography of the choroid in normal eyes. Am J Ophthalmol. (2009) 1475:811–5. 10.1016/j.ajo.2008.12.008 19232559

[58] Touhami S Philippakis E Mrejen S Couturier A Casteran C Levent P. Topographic variations of choroidal thickness in healthy eyes on swept-source optical coherence tomography. Invest Ophthalmol Vis Sci. (2020) 613:38. 10.1167/iovs.61.3.38 32196096 PMC7401446

[59] Ikuno Y Kawaguchi K Nouchi T Yasuno Y. Choroidal thickness in healthy Japanese subjects. Invest Ophthalmol Vis. (2010) 514:2173. 10.1167/iovs.09-4383 19892874

[60] Ruiz-Moreno JM Flores-Moreno I Lugo F Ruiz-Medrano J Montero JA Akiba M. Macular choroidal thickness in normal pediatric population measured by swept-source optical coherence tomography. Invest Ophthalmol Vis Sci. (2012) 54:353–9. 10.1167/iovs.12-10863 23249703

[61] Tan C Ouyang Y Ruiz H Sadda S. Diurnal variation of choroidal thickness in normal, healthy subjects measured by spectral domain optical coherence tomography. Invest Ophthalmol Vis Sci. (2012) 531:261–6. 10.1167/iovs.11-8782 22167095

[62] Ding X Li J Zeng J Ma W Liu R Li T. Choroidal thickness in healthy Chinese subjects. Invest Ophthalmol Vis Sci. (2011) 5213:9555–60. 10.1167/iovs.11-8076 22058342

[63] Bhayana A Kumar V Tayade A Chandra M Chandra P Kumar A. Choroidal thickness in normal Indian eyes using swept-source optical coherence tomography. Indian J Ophthalmol. (2019) 67:252–5. 10.4103/ijo.IJO_668_18 30672480 PMC6376823

[64] Wakatsuki Y Shinojima A Kawamura A Yuzawa M. Correlation of aging and segmental choroidal thickness measurement using swept source optical coherence tomography in healthy eyes. PLoS ONE. (2015) 1012:e0144156. 10.1371/journal.pone.0144156 26632821 PMC4669163

[65] Hirata M Tsujikawa A Matsumoto A Hangai M Ooto S Yamashiro K. Macular choroidal thickness and volume in normal subjects measured by swept-source optical coherence tomography. Invest Ophthalmol Vis Sci. (2011) 528:4971–8. 10.1167/iovs.11-7729 21622704

[66] Lee S Lingham G Alonso-Caneiro D Chen F Yazar S Hewitt A. Choroidal thickness in young adults and its association with visual acuity. Am J Ophthalmol. (2020) 214:40–51. 10.1016/j.ajo.2020.02.012 32112771

[67] Matsuo Y Sakamoto T Yamashita T Tomita M Shirasawa M Terasaki H. Comparisons of choroidal thickness of normal eyes obtained by two different spectral-domain OCT instruments and one swept-source OCT instrument. Invest Ophthalmol Vis Sci. (2013) 5412:7630–6. 10.1167/iovs.13-13135 24168999

[68] Adhi M Liu J Qavi A Grulkowski I Lu C Mohler K. Choroidal analysis in healthy eyes using swept-source optical coherence tomography compared to spectral domain optical coherence tomography. Am J Ophthalmol. (2014) 1576:1272–81.e1. 10.1016/j.ajo.2014.02.034 24561169

[69] Copete S Flores-Moreno I Montero J Duker J Ruiz-Moreno J. Direct comparison of spectral-domain and swept-source OCT in the measurement of choroidal thickness in normal eyes. Br J Ophthalmol. (2014) 983:334–8. 10.1136/bjophthalmol-2013-303904 24288394

[70] Castro-Navarro V Behar-Cohen F Chang W Joussen A Lai T Navarro R. Pachychoroid: current concepts on clinical features and pathogenesis. Graefes Arch Clin Exp Ophthalmol. (2020) 2596:1385–400. 10.1007/s00417-020-04940-0 33057904 PMC8166704

[71] Chirco K Sohn E Stone E Tucker B Mullins R. Structural and molecular changes in the aging choroid: implications for age-related macular degeneration. Eye. (2017) 311:10–25. 10.1038/eye.2016.216 27716746 PMC5233940

[72] Prünte C Flammer J. Choroidal capillary and venous congestion in central serous chorioretinopathy. Am J Ophthalmol. (1996) 1211:26–34. 10.1016/S0002-9394(14)70531-8 8554078

[73] Borooah S Sim P Phatak S Moraes G Wu C Cheung C. Pachychoroid spectrum disease. Acta Ophthalmol. (2020) 99:e806–22. 10.1111/aos.14683 33258304

[74] Jordan-Yu J Teo K Chakravarthy U Gan A Tan A Cheong K. polypoidal choroidal vasculopathy features vary according to subfoveal choroidal thickness. Retina. (2021) 415:1084–93. 10.1097/IAE.0000000000002966 32858669

[75] Maruko I Iida T Sugano Y Ojima A Ogasawara M Spaide R. Subfoveal choroidal thickness after treatment of central serous chorioretinopathy. Ophthalmology. (2010) 1179:1792–9. 10.1016/j.ophtha.2010.01.023 20472289

[76] Alovisi C Piccolino F Nassisi M Eandi CM. Choroidal structure after half-dose photodynamic therapy in chronic central serous chorioretinopathy. J Clin Med. (2020) 9:2734. 10.3390/jcm9092734 32847076 PMC7563121

[77] Kim Y Ryoo N Woo S Park K. Choroidal thickness changes after photodynamic therapy and recurrence of chronic central serous chorioretinopathy. Am J Ophthalmol. (2015) 1601:72–84.e71. 10.1016/j.ajo.2015.04.011 25887629

[78] Kim D Joe S Yang H Lee J Kim J Yoon Y. Subfoveal choroidal thickness changes in treated idiopathic central serous chorioretinopathy and their association with recurrence. Retina. (2015) 359:1867–74. 10.1097/IAE.0000000000000557 25946693

[79] Annesley WH Jr. Peripheral exudative hemorrhagic chorioretinopathy. Trans Am Ophthalmol Soc. (1980) 78:321–64 7257064 PMC1312148

[80] Mantel I Schalenbourg A Zografos L. Peripheral exudative hemorrhagic chorioretinopathy: polypoidal choroidal vasculopathy and hemodynamic modifications. Am J Ophthalmol. (2012) 1535:910–22.e2. 10.1016/j.ajo.2011.10.017 22310077

[81] Kitagawa Y Shimada H Kawamura A Tanaka K Mori R Onoe H. A case of bilateral pachychoroid disease: polypoidal choroidal vasculopathy in one eye and peripheral exudative hemorrhagic chorioretinopathy in contralateral eye. BMC Ophthalmol. (2021) 21:320. 10.1186/s12886-021-02067-2 34481477 PMC8418046

[82] Shroff D Sharma M Chhablani J Gupta P Gupta C Shroff C. Peripheral exudative hemorrhagic chorioretinopathy-a new addition to the spectrum of pachychoroid disease? Retina. (2021) 417:1518–25. 10.1097/IAE.0000000000003063 33315818

[83] Takahashi H Takase H Ishizuka A Miyanaga M Kawaguchi T Ohno-Matsui K. Choroidal thickness in convalescent vogt-koyanagi-harada disease. Retina. (2014) 344:775–80. 10.1097/IAE.0b013e3182a6b3f6 23979311

[84] Fong A Li K Wong D. Choroidal evaluation using enhanced depth imaging spectral-domain optical coherence tomography in Vogt-Koyanagi-Harada disease. Retina. (2011) 313:502–9. 10.1097/IAE.0b013e3182083beb 21336069

[85] Nakayama M Keino H Okada A Watanabe T Taki W Inoue M. Enhanced depth imaging optical coherence tomography of the choroid in Vogt-Koyanagi-Harada disease. Retina. (2012) 3210:2061–9. 10.1097/IAE.0b013e318256205a 23095726

[86] Jap A Chee S. The role of enhanced depth imaging optical coherence tomography in chronic Vogt-Koyanagi-Harada disease. Br J Ophthalmol. (2017) 1012:186–9. 10.1136/bjophthalmol-2015-308091 27048179

[87] Jonas J Forster T Steinmetz P Schlichtenbrede F Harder B. Choroidal thickness in age-related macular degeneration. Retina. (2014) 346:1149–55. 10.1097/IAE.0000000000000035 24220257

[88] Yiu G Chiu S Petrou P Stinnett S Sarin N Farsiu S. Relationship of central choroidal thickness with age-related macular degeneration status. Am J Ophthalmol. (2015) 1594:617–26. 10.1016/j.ajo.2014.12.010 25526948

[89] Sigler E Randolph J. Comparison of macular choroidal thickness among patients older than age 65 with early atrophic age-related macular degeneration and normals. Invest Ophthalmol Vis Sci. (2013) 549:6307–13. 10.1167/iovs.13-12653 23982844

[90] Park J Kang M Kim B Chung K Sim H Lee S. Topographic changes in choroidal thickness in age-related macular degeneration during the development of active choroidal neovascularization. Retina. (2020) 412:409–22. 10.1097/IAE.0000000000002845 32453064

[91] Kumar J Wai K Ehlers J Singh R Rachitskaya A. Subfoveal choroidal thickness as a prognostic factor in exudative age-related macular degeneration. Br J Ophthalmol. (2019) 1037:918–21. 10.1136/bjophthalmol-2018-312625 30150279

[92] Xie J Chen Q Yu J Zhou H He J Wang W. Morphologic features of myopic choroidal neovascularization in pathologic myopia on swept-source optical coherence tomography. Front Med. (2020) 7:615902. 10.3389/fmed.2020.615902 33425961 PMC7785753

[93] Wu Q Chen Q Lin B Huang S Wang Y Zhang L. Relationships among retinal/choroidal thickness, retinal microvascular network and visual field in high myopia. Acta Ophthalmol. (2020) 98:e709–14. 10.1111/aos.14372 32030900

[94] Fledelius H Jacobsen N Li X Goldschmidt E. Choroidal thickness at age 66 years in the Danish high myopia study cohort 1948 compared with follow-up data on visual acuity over 40 years: a clinical update adding spectral domain optical coherence tomography. Acta Ophthalmol. (2018) 961:46–50. 10.1111/aos.13659 29356366

[95] Li Z Wang W Liu R Wang D Zhang J Xiao O. Choroidal thickness predicts progression of myopic maculopathy in high myopes: a 2-year longitudinal study. Br J Ophthalmol. (2020) 1–7. 10.1136/bjophthalmol-2020-316866 32972914

[96] Reibaldi M Boscia F Avitabile T Uva M Russo V Zagari M. Enhanced depth imaging optical coherence tomography of the choroid in idiopathic macular hole: a cross-sectional prospective study. Am J Ophthalmol. (2011) 1511:112–7.e2. 10.1016/j.ajo.2010.07.004 20970113

[97] Sogawa K Nagaoka T Takahashi A Tanano I Tani T Ishibazawa A. Relationship between choroidal thickness and choroidal circulation in healthy young subjects. Am J Ophthalmol. (2012) 1536:1129–32.e1. 10.1016/j.ajo.2011.11.005 22310083

[98] Gass J. Reappraisal of biomicroscopic classification of stages of development of a macular hole. Am J Ophthalmol. (1995) 1196:752–9. 10.1016/S0002-9394(14)72781-3 7785690

[99] Balmforth C van Bragt J Ruijs T Cameron J Kimmitt R Moorhouse R. Chorioretinal thinning in chronic kidney disease links to inflammation and endothelial dysfunction. JCI Insight. (2016) 1:e89173. 10.1172/jci.insight.89173 27942587 PMC5135281

[100] Gifford F Moroni F Farrah T Hetherington K MacGillivray T Hayes P. The Eye as a non-invasive window to the microcirculation in liver cirrhosis: a prospective pilot study. J Clin Med. (2020) 910:3332. 10.3390/jcm9103332 33080821 PMC7603064

[101] Salobrar-Garcia E Méndez-Hernández C Hoz R Ramírez A López-Cuenca I Fernández-Albarral J. Ocular vascular changes in mild alzheimer's disease patients: foveal avascular zone, choroidal thickness, and ONH hemoglobin analysis. J Personalized Med. (2020) 104:231. 10.3390/jpm10040231 33203157 PMC7712569

[102] Mansouri K Medeiros F Tatham A Marchase N Weinreb R. Evaluation of retinal and choroidal thickness by swept-source optical coherence tomography: repeatability and assessment of artifacts. Am J Ophthalmol. (2014) 1575:1022–32. 10.1016/j.ajo.2014.02.008 24531020 PMC5596889

